# Identification of Novel Human Dipeptidyl Peptidase-IV Inhibitors of Natural Origin (Part II): *In Silico* Prediction in Antidiabetic Extracts

**DOI:** 10.1371/journal.pone.0044972

**Published:** 2012-09-21

**Authors:** Laura Guasch, Esther Sala, María José Ojeda, Cristina Valls, Cinta Bladé, Miquel Mulero, Mayte Blay, Anna Ardévol, Santiago Garcia-Vallvé, Gerard Pujadas

**Affiliations:** 1 Grup de Recerca en Nutrigenòmica, Departament de Bioquímica i Biotecnologia, Universitat Rovira i Virgili, Tarragona, Catalonia, Spain; 2 Centre Tecnològic de Nutrició i Salut (CTNS), TECNIO, CEICS, Reus, Catalonia, Spain; University of South Florida College of Medicine, United States of America

## Abstract

**Background:**

Natural extracts play an important role in traditional medicines for the treatment of diabetes mellitus and are also an essential resource for new drug discovery. Dipeptidyl peptidase IV (DPP-IV) inhibitors are potential candidates for the treatment of type 2 diabetes mellitus, and the effectiveness of certain antidiabetic extracts of natural origin could be, at least partially, explained by the inhibition of DPP-IV.

**Methodology/Principal Findings:**

Using an initial set of 29,779 natural products that are annotated with their natural source and an experimentally validated virtual screening procedure previously developed in our lab (Guasch et al.; 2012) [1], we have predicted 12 potential DPP-IV inhibitors from 12 different plant extracts that are known to have antidiabetic activity. Seven of these molecules are identical or similar to molecules with described antidiabetic activity (although their role as DPP-IV inhibitors has not been suggested as an explanation for their bioactivity). Therefore, it is plausible that these 12 molecules could be responsible, at least in part, for the antidiabetic activity of these extracts through their inhibitory effect on DPP-IV. In addition, we also identified as potential DPP-IV inhibitors 6 molecules from 6 different plants with no described antidiabetic activity but that share the same *genus* as plants with known antidiabetic properties. Moreover, none of the 18 molecules that we predicted as DPP-IV inhibitors exhibits chemical similarity with a group of 2,342 known DPP-IV inhibitors.

**Conclusions/Significance:**

Our study identified 18 potential DPP-IV inhibitors in 18 different plant extracts (12 of these plants have known antidiabetic properties, whereas, for the remaining 6, antidiabetic activity has been reported for other plant species from the same *genus*). Moreover, none of the 18 molecules exhibits chemical similarity with a large group of known DPP-IV inhibitors.

## Introduction

Medical plants play an important role in the management of type 2 diabetes mellitus (T2DM) by delaying the development of diabetic complications and correcting metabolic abnormalities [2]. Traditional plant-based remedies have been and are being used by T2DM patients around the world (*e.g.*, patients belonging to the Chinese [3], Indian [4] and Mexican [5] populations), and many scientific studies have confirmed the benefits of medicinal plants with hypoglycemic effects on these patients [6]–[9]. Furthermore, during the past few years, some of the new bioactive drugs isolated from hypoglycemic plants have been demonstrated to have antidiabetic activity with greater efficacy than synthetic oral hypoglycemic agents used in clinical therapy regimens [10].

The most commonly studied hypoglycemic plants are *Opuntia streptacantha*, *Trigonella foenum-graecum*, *Momordica charantia*, *Ficus bengalensis*, *Polygala senega* and *Gymnema sylvestre* [10]. Despite their long tradition of use worldwide, few of these plants have been tested in modern, large-scale, clinical-type trials to determine their efficacies. However, it is clear that more research needs to be undertaken on these and other medicinal plants with hypoglycemic effects because, in most cases, the bioactive compounds and their modes of action still remain unclear.

Numerous mechanisms of antidiabetic action have been proposed for extracts of the previous mentioned plants, some of them relate to their ability to stimulate insulin secretion [11]. Regarding the stimulation of insulin secretion, one target of interest for the antidiabetic action of these extracts is the serine protease dipeptidyl peptidase-IV (DPP-IV; EC 3.4.14.5) because the inhibition of DPP-IV has been shown to be an appropriate treatment for T2DM [12]. DPP-IV specifically removes N-terminal dipeptides from substrates containing proline or alanine as the second residue, transforming them into inactive or even antagonistic species. The most important substrates of DPP-IV are incretins, such as glucagon-like peptide-1 (GLP-1) and glucose-dependent insulinotropic polypeptide (GIP), which stimulates insulin secretion [13]. Incretin hormones are intestinal hormones that are released in response to nutrient ingestion and that potentiate the glucose-induced insulin response. Therefore, GLP-1 stimulates insulin biosynthesis and secretion, reduces glucagon release, slows gastric emptying, reduces appetite, and stimulates the regeneration and differentiation of islet B-cells [14]. On the other hand, GIP is involved in glucose metabolism by enhancing insulin secretion [15]. Both peptides have short half-lives because of their rapid degradation by DPP-IV. Therefore, inhibiting DPP-IV prolongs the action of GLP-1 and GIP, which, in turn, improves glucose homeostasis with a low risk of hypoglycemia [12].

The first DPP-IV inhibitor on the market was sitagliptin (by Merck & Co.) [16], which was followed by the structurally similar vildagliptin (by Novartis) [17] and saxagliptin (by Bristol-Myers Squibb and AstraZeneca) [18]. The efficacy and safety profile of DPP-IV inhibitors have been promising and advantageous to date. In contrast to sulfonylureas and other antidiabetic drugs, DPP-IV inhibitors do not have an intrinsic risk of inducing hypoglycemia, and they are body-weight neutral. Their tolerability profile is good, and no specific adverse reactions have been reported [12].

The DPP-IV binding site is highly druggable in the sense that tight, specific binding to the enzyme can be achieved with small molecules with drug-like physicochemical properties [19], [20]. The two key binding-site areas for the intermolecular interaction of DPP-IV and reversible inhibitors of non-peptide nature are the lipophilic S1 pocket (formed by Tyr631, Val656, Trp659, Tyr662, Tyr666 and Val711) and the negatively charged Glu205/206 pair [20]. We have recently used coordinates from complexes between DPP-IV and potent reversible inhibitors of non-peptide nature to derive a structure-based common pharmacophore that defines a common background for DPP-IV inhibition [1]. This pharmacophore is part of a virtual screening (VS) workflow that also includes protein-ligand docking studies and shape and electrostatic-potential comparisons [1]. We have applied this VS workflow to the 89,425 molecules present in the natural products subset of the ZINC database (http://wiki.bkslab.org/index.php/Natural_products_database), and we found that 446 of these molecules would inhibit DPP-IV with good ADMET properties. Notably, when these 446 molecules were merged with 2,342 known DPP-IV inhibitors, and the resulting set was classified into 50 clusters according to chemical similarity, there were 12 clusters that contained only natural products for which no DPP-IV inhibitory activity has been previously reported. Nine molecules from 7 of these 12 clusters were then selected for *in vitro* activity testing, and 7 out of the 9 molecules were shown to inhibit DPP-IV (the remaining two molecules could not be solubilized, preventing the evaluation of their DPP-IV inhibitory activity) [1].

The goal of the present work was to identify natural extracts with known antidiabetic activity that contain at least one molecule that we predict to be a DPP-IV inhibitor through a slightly modified version of the VS workflow described above [1]. Therefore, in this study, we provide new information about the active molecules in some natural extracts with antidiabetic properties and suggest that, at least in part, the mode of action of these molecules involves stimulating insulin secretion through the inhibition of DPP-IV. We also provide a list of plants with no previously described antidiabetic activity that may contain DPP-IV inhibitors and that are related to plants with known antidiabetic activity. These plants represent a new source of potential antidiabetic extracts. In addition, the new DPP-IV inhibitors that we identified are chemically different from known DPP-IV inhibitors, and therefore, they could be used as lead-hopping candidates for the development of new antidiabetic drugs.

## Results and Discussion

### Virtual Screening Description and Application

We used a slightly modified version of a VS workflow that was previously developed and experimentally validated [1] to identify DPP-IV inhibitors in a large in-house database of natural products (NPs) annotated with their natural source.

**Figure 1:**
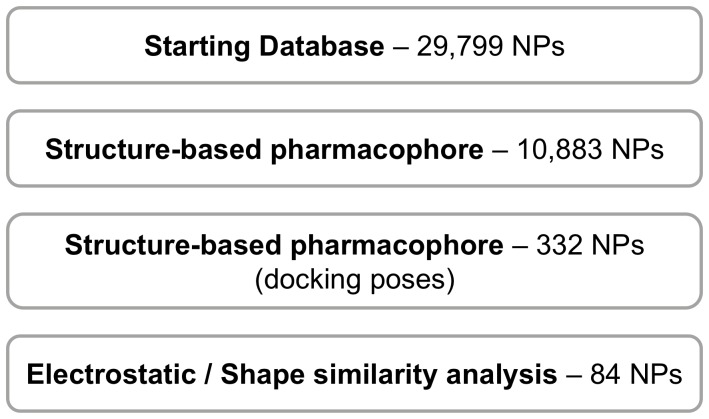
The VS workflow used in the present work. The number beside each VS step is the number of molecules that *survived* that step.

The VS workflow (see Figure 1) consisted of several sequential steps in which the output molecules of one step were the input molecules for the next step and so on. Central in this workflow is one structure-based common pharmacophore that captures the key intermolecular interactions needed for drugs to inhibit DPP-IV; this pharmacophore is formed by 2 mandatory sites (*i.e.*, one positive/donor and one hydrophobic/aromatic ring) and 5 optional sties (*i.e.*, two hydrogen-bond acceptors and three hydrophobic/aromatic ring sites). Both mandatory sites interact with crucial molecular anchors for DPP-IV inhibition (*i.e.*, the hydrophobic/aromatic ring interacts with the hydrophobic S1 pocket at the DPP-IV binding site, and the positive/donor site interacts with the Glu205/Glu206 dyad [20]). Briefly, the VS workflow consists of (1) comparing ligand conformers to the common pharmacophore by allowing reorientation of the conformers to determine if they match the pharmacophore; (2) using ligands with at least one hit in the previous filter in a protein-ligand rigid-docking study and docking them onto the ligand binding site of the DPP-IV conformation present in the 3C45 PDB file; (3) comparing the resulting docking conformations to the structure-based common pharmacophore without reorienting the poses; and (4) submitting the poses that were hits in the previous filter to a shape and electrostatic-potential comparison with the experimental pose of the DPP-IV inhibitor in the PDB file 3C45 (the previously developed VS workflow [1] was altered for the current work to use slightly lower threshold values for the electrostatic and shape comparisons).

This VS protocol was applied to an in-house database of 29,779 NPs with appropriate ADME/Tox properties. The first filter found that 10,883 molecules in our database have at least one conformer that after proper reorientation, matches the pharmacophore (see Figure 1). Only 332 out of these 10,883 molecules have docked conformations that without reorientation, are able to match the pharmacophore (see Figure 1). This reduction is useful because it discards those molecules that are predicted to bind in a non-productive way to the DPP-IV binding site. Finally, the later filter (*i.e.*, electrostatic and shape similarity screening) aims to smooth differences in chemical structures and translate them into criteria important for their intermolecular interactions with the ligand-binding site. This filter has been reported to be a valuable VS tool for the discovery of novel scaffolds [21], and in our case, it was applied to rescore the 332 hits that survived the previous filter. Consequently, only those molecules that had at least one docked pose that met the following conditions in the comparisons made by EON were predicted to inhibit DPP-IV: (a) *ET_pb* ≥0.468 and (b) ST ≥0.237. Interestingly, the fact that DPP-IV inhibitors (a) have a significant positive electrostatic potential in the region that interacts with the Glu205/Glu206 dyad (see Figure 1) and (b) that this ligand area matches the mandatory positive/donor site justifies the dominance of the electrostatic contribution over the shape contribution in the selected thresholds. Finally, the VS workflow identified 84 molecules with potential DPP-IV inhibitory activity (see Figure 1).

### Virtual Screening Hits in Natural Extracts with Known Antidiabetic Activity

According to the information available in our in-house NPs database, the 84 molecules that were predicted by the VS workflow as potential DPP-IV inhibitors have been isolated from 95 different natural sources. Interestingly, a systematic bibliographic search of PubMed (http://www.pubmed.org) revealed that the extracts of 12 out of these 95 natural sources have been reported to exhibit antidiabetic activity (see Table S1). Moreover, among these 12 sources we found 12 VS hits that may, through their role as DPP-IV inhibitors, contribute to the observed antidiabetic activity of their corresponding extracts (see Table S1). In fact, a search using SciFinder (http://www.cas.org/products/sfacad) revealed that 6 out of these 12 natural compounds correspond (or are similar in chemical structure) to molecules with known antidiabetic properties (see Table S1). This finding further validates our methodology (in addition to the experimental validation of the original VS workflow [1]) and suggests that the mode of action of these molecules could involve DPP-IV inhibition. The remaining 6 natural compounds not previously reported to have antidiabetic properties represent new molecules that may also exhibit this bioactivity. In the next paragraphs, the most significant compounds found in these 12 antidiabetic extracts are discussed:

- N-Nororientaline (CAS number 29079-44-5, see Table S1) has been isolated from five plant species from *genus Erythrina* (*i.e.*, *Erythrina variegata* [22], *Erythrina crystagalli, Erythrina indica, Erythrina poeppigiana* [22], and *Erythrina arborescens*) and was identified as a DPP-IV inhibitor by our VS procedure. Extracts from *Erythrina variegata*, *Erythrina senegalensis*, *Erythrina addisoniae*, *Erythrina abyssinica*, and *Erythrina mildbraedii* are reported to enhance antidiabetic activity [23]–[27]. Isoprenylated flavonoids isolated from *Erythrina mildbraedii* and prenylflavonoids isolated from the *Erythrina senegalensis* roots have been described as inhibitors of two other proteins frequently targeted in T2DM treatment (*i.e.*, protein tyrosine phosphatase-1B [25], [26] for the former class of molecules and acyl CoA:diacylglycerol acyltransferase [24] for the latter class). All of these flavonoid compounds are chemically related to our hit. Therefore, the antidiabetic action of extracts from these plants may be the result of more than one bioactive component and mode of action.
- Tecostamine, which is found in *Tecoma stans*, has hypoglycemic properties similar to those of tecomine [28]. Our results therefore suggest that the hypoglycemic properties of tecostamine could be mediated by the inhibition of DPP-IV. In addition, the *Tecoma stans* aqueous extract posses at least four antidiabetic-related activities (*i.e.*, intestinal á-glucosidase inhibition, post-prandial antihyperglycemic, hypocholesterolemic and hypotriglyceridemic effects). Therefore, although it is possible that most of these activities are exerted by the phenolic compounds present in the *Tecoma stans* aqueous extract, bioguided studies are necessary to confirm this hypothesis [29].
- Epinephrine (also known as adrenaline), which is found in *Scoparia dulcis*, has been reported to improve hypoglycemia [30]. *Scoparia dulcis* has been described as a folk-medicinal plant and has been traditionally used as a remedy for diabetes mellitus in India and for hypertension in Taiwan [31]. From Indian *Scoparia dulcis*, an antidiabetic compound named amellin was also isolated and characterized by Nath [32].
- From the same family as epinephrine, two additional compounds were predicted by our VS to be potential DPP-IV inhibitors. These compounds are (+)-pseudoephedrine (CAS number 90-82-4) and (−)-ephedrine (CAS number 299-42-3), and both have also been reported to have hypoglycemic activity [33]. Both molecules are found in several *Ephedra* species (*Ephedra alata* [34], *Ephedra distachya* [35], *Ephedra equisetina* [36], *Ephedra gerardiana* [35], *Ephedra shennungiana* [37], *Ephedra sinica*, *Ephedra vulgaris* and *Ephedra pflanze*). However, *Ephedra distachya* and *Ephedra alata* are the only species reported to have antidiabetic properties [33], [38]; therefore, we proposed that the remaining *Ephedra* species may be new sources of antidiabetic extracts.
- The molecules ajmaline and isosandwichine, which are enantiomers with the same CAS number, 509-37-5, are found in several *Rauwolfia* species. *Rauwolfia vomitoria* has been investigated for the content of alkaloids, especially those alkaloids with hypotensive and anti-inflammatory properties [39] in addition to the antidiabetic properties. *Rauwolfia serpentina* is also used as antidiabetic extract [40]. The remaining species that contain these molecules, such as *Rauwolfia canescens* [41], *Rauwolfia degeneri* [42], *Rauwolfia densiflora* [43], *Rauwolfia heterophylla* [44], *Rauwolfia indecora*, *Rauwolfia obscura* [45] and *Rauwolfia tetraphylla* [42], are putative antidiabetic extracts.
- Serpinine (CAS number 509-38-6) is isolated from *Vinca major* and several *Rauwolfia* species (*Rauwolfia obscura* [45], *Rauwolfia tetraphylla*, *Rauwolfia serpentina* [46] and *Rauwolfia sellowii* [1]), belongs to the same cluster as ajmaline and isosandwichine (*i.e.*, cluster 78; see Table S1). Therefore, they share similar chemical structures and natural sources. Moreover, *Vinca major* organic leaf extract strongly stimulates glucose utilization [47].
- One interesting hit predicted to be a DPP-IV inhibitor is an epicatechin derivate that is found in *Vitis vinifera*. An antihyperglycemic effect in streptozotocin-induced diabetic rats and insulinomimetic activity in insulin-sensitive cell lines have been described for grape seed procyanidin extracts (GSPE) [48]. In addition, it has been demonstrated that oligomeric procyanidins from GSPE activate the insulin receptor by interacting with and inducing the phosphorylation of the insulin receptor and that this interaction leads to increased glucose uptake [49]. Moreover, several epicatechin derivates have been reported to have antidiabetic properties (the most studied of which is epigallocatechin gallate [50]). Some findings demonstrate that epigallocatechin gallate may be a novel, plant-derived compound capable of reducing the risk of type 1 diabetes [51]. Therefore, the DPP-IV inhibition induced by this epicatechin derivate may contribute to the antihyperglycemic effect of GSPE [48].
- The remaining 3 molecules predicted to be DPP-IV inhibitors through our VS workflow and that are found in extracts with described antidiabetic properties are hydroxysmirnovine from *Galega orientalis* [52], (−)-halosaline (CAS number 26648-71-5) from *Haloxylon salicornicum* [53] and isochanoclavin-(I) (CAS number 1150-44-3) from *Pennisetum typhoideum* [54] (see Table S1). Our results suggest that these molecules could be DPP-IV inhibitors and that extracts containing these molecules could potentiate the glucose-induced insulin response by prolonging the half-lives of GLP-1 and GIP incretins, due to the inhibition of DPP-IV. This information is novel and relevant, as no mechanism that explains the antidiabetic properties of these extracts has been previously suggested.

### Virtual Screening Hits in Natural Extracts with No Described Antidiabetic Activity

Taking into account that extracts from closely related species of the same *genus* may share a high number of components, we also determined if any of our VS hits that were isolated from plants with no described antidiabetic activity belong to the same *genus* as species with known antidiabetic properties. We identified 6 molecules isolated from 6 different plants, *Aconitum japonicum*, *Ervatamia officinalis*, *Solanum nudum*, *Solanum sodomaeum*, *Stephania cepharantha* and *Tabernaemontana eglandulosa* (see Table S2), that meet these criteria. The related species with described antidiabetic properties are *Aconitum carmichaelii* [33], *Aconitum moschatum* [2], *Aconitum violaceum* [2], *Ervatamia microphylla* [55], *Solanum lycocarpum* [56], *Solanum nigrum* [57], *Solanum xanthocarpum* [58], *Stephania hernandifolia* [59], *Stephania glabra* [60], *Stephania tetrandra* [61], and *Tabernaemontana divaricata* [55]. Therefore, it is plausible to hypothesize that these 6 plants could also have antidiabetic properties mediated, at least partially, by the inhibition of DPP-IV.

### Finding New Scaffolds of Natural Origin for DPP-IV Inhibitors

The 18 molecules in Tables S1 and S2 that were predicted to be DPP-IV inhibitors are of interest. To quantify the number of new scaffolds for DPP-IV inhibitors that were identified in our study, we merged the 18 VS hits with 2,342 known DPP-IV inhibitors, and the resulting set was classified according to structural similarity into 99 clusters (results not shown). Interestingly, the 18 hits were classified in 13 clusters (see Tables S1 and S2) that do not contain known DPP-IV inhibitors (results not shown). Thus, these 18 predicted DPP-IV inhibitors correspond to 13 different chemical scaffolds that are unrelated to those present in known DPP-IV inhibitors, and, consequently, these new scaffolds could be used either in lead-hopping experiments to identify new DPP-IV inhibitors or in structure-activity studies to identify natural-product derivates with stringer DPP-IV inhibition activity than the original NPs from which they are derived.

### Conclusions

In a previous study [1], we developed a VS workflow that was able to distinguish successfully molecules that inhibit DPP-IV and molecules that do not inhibit this enzyme. We experimentally demonstrated that our VS protocol was able to identify DPP-IV inhibitors that **(a)** were not structurally related to any known molecule that inhibits DPP-IV and **(b)** have never been reported to have antidiabetic activity. In the present work, we applied a slightly modified version of this VS workflow to an in-house database of 29,779 NPs annotated with their corresponding natural source(s). From this initial set of NPs, our VS procedure identified as potential DPP-IV inhibitors 84 hit molecules that have been isolated from 95 different natural sources. Interestingly, after an exhaustive bibliographic search, our results demonstrate that we are able to predict (a) 12 DPP-IV inhibitors that are present in 12 plant extracts with known antidiabetic activity and (b) 6 DPP-IV inhibitors that are present in 6 different plants species with no described antidiabetic activity but that share the same *genus* as plants with known antidiabetic properties (consequently, it could be suggested that these plants represent a potential new source of antidiabetic extracts). Moreover, none of these 18 hits exhibits chemical similarity with 2,342 known DPP-IV inhibitors, and, therefore, it is expected that a significant number of these hits could be lead-hopping candidates for the development of new DPP-IV inhibitors. At this point, it is also interesting to note that the analysis of the chemical structures of these 18 NP hits revealed that the majority of them are alkaloids containing basic nitrogen atoms (essential for proper interaction with the Glu205/Glu206 dyad). Moreover, our results provide a new hypothesis about the mechanisms by which, at least partially, these 12 extracts exert their antidiabetic effects (*i.e.*, improving glucose homeostasis by prolonging the activity of GLP-1 and GIP through DPP-IV inhibition).

Lastly, we predicted that there are 77 other sources with no described antidiabetic activity that contain at least one VS hit. Consequently, our work opens the door to the discovery of new antidiabetic extracts of natural origin that could be of use, for example, in the design of functional foods aimed at preventing/treating T2DM. Therefore, the characterization of such extracts merits further attention, and such work is currently underway.

## Methods

### Initial Dataset of Natural Compounds Used

The database of NPs that was screened by the VS workflow contains 29,779 NPs from different origins (*e.g.*, plants, and fungi) with appropriate ADME/Tox properties and no chiral ambiguities. An important characteristic of this database is that each molecule is annotated with (a) the natural sources from which it has been obtained and (b) the bibliographic references that describe how to extract the relevant molecule from each natural source. The 3D structures of the molecules in this NP database were processed with LigPrep v2.3 (Schrödinger LLC., Portland, USA; http://www.schrodinger.com) using the following parameters: (a) the force field used was OPLS 2005; (b) all possible ionization states at pH 7.0±2.0 were generated with Ionizer; (c) the desalt option was activated; (d) tautomers were generated for all ionization states at pH 7.0±2.0; (e) chiralities were determined from the 3D structure; and (f) one low-energy ring conformation per ligand was generated. Conformations and sites for the resulting ligand structures were determined during the generation of the corresponding Phase [62] databases with the ***Generate Phase Database*** graphic front-end. Default parameter values were used during this conformer generation process, with the exception of the maximum number of conformers per structure, which was increased from 100 (the default value) to 200. The conformer sites were generated with definitions made by adding to the default built-in Phase definitions the ability to consider aromatic rings as hydrophobic groups.

### Virtual Screening Workflow

The VS workflow used in this work is the same as that described previously [1], except that the conditions of the last filter (*i.e.*, the shape and electrostatic potential comparison) were slightly different, as outlined below.

The VS protocol used a structure-based pharmacophore that was built by (1) selecting from the PDB those reliable complexes of human DPP-IV and potent inhibitors of non-peptide nature (*i.e.*, IC_50_≤10 nM) that bind reversibly to the enzyme; (2) using their corresponding DPP-IV coordinates to guide the superposition of the remaining PDB files (the resulting re-oriented coordinates for these PDB files were also used in the pharmacophore-based searches, protein-ligand docking studies and shape and electrostatic-potential comparisons of the VS workflow); (3) using the resulting coordinates to derive the corresponding energetic structure-based pharmacophores; and (4) building the common structure-based pharmacophore for reversible DPP-IV inhibition by prioritizing energetically favorable features over energetically weaker ones. The resulting pharmacophore consists of two compulsory sites (one positive/donor and one hydrophobic/aromatic ring) and five optional sites (*i.e.*, two acceptor sites and three hydrophobic/aromatic ring sites) and was completed with receptor-based excluded volumes that schematically represent the location of the DPP-IV residues that form the binding pocket in the PDB file 3C45.

The first step of the VS workflow uses the common structure-based pharmacophore to screen the conformer database with Phase v3.1 (Schrödinger LLC., Portland, USA; http://www.schrodinger.com) and allows the reorientation of the conformers to determine if they match the pharmacophore. Only those ligands with at least one conformer that matches the two compulsory sites of the common pharmacophore and at least one of the optional sites (without sterically colliding with the excluded volumes) *survive* this VS step. These ligands were docked onto the binding pocket of the PDB file 3C45 with eHiTS v2009 (SimBioSys Inc., Toronto, Canada; http://www.simbiosys.ca/ehits) [63] using default docking conditions, with the exception of the length of the sides of the cubic box encompassing the DPP-IV binding site, which was increased from 10 Å to 15 Å. Next, the resulting docked poses were again filtered with Phase through the common pharmacophore using the same filtering conditions as in the first Phase run (with the exception that no reorientation of the docked poses was allowed during the search). Thus, only docking poses compatible with the pharmacophore *survived* this filter. Finally, in the last step of the VS protocol, the poses that were hits in this second pharmacophore screen were submitted to a shape and electrostatic potential comparison with the DPP-IV inhibitor present in the 3C45 PDB file to rescore the hits. This comparison was completed with EON v2.0.1 (OpenEye Scientific Software, Inc., Santa Fe, New Mexico, USA; http://www.eyesopen.com) and used the Electrostatic Tanimoto combo (ET_combo) score as the similarity criterion. The ET_combo score is the sum of two calculations: (a) the Shape Tanimoto (ST) score, which is a quantitative measure of three-dimensional overlap (where 1 corresponds to a perfect overlap; *i.e.* the same shape), and (b) the Poisson-Boltzmann Electrostatic Tanimoto (ET_pb) score, which compares the electrostatic potential of two small molecules and ranges from 1 (identical potential) to a negative value that results from the overlap of positive and negative charges. In this work, we determined the EON threshold scores by comparing the inhibitor in 3C45 with the inhibitors found in a group of 24 PDB complexes of DPP-IV and reversible drugs that inhibit this protein (*i.e.*, 1N1M, 20GZ, 2FJP, 2HHA, 2I78, 2IIT, 2IIV, 2OLE, 2ONC, 2OQI, 2OQV, 2QOE, 2QT9, 2QTB, 2RGU, 2RIP, 3C43, 3CCC, 3D4L, 3F8S, 3H0C, 3HAB, and 3HAC). This comparison of the experimental poses of DPP-IV inhibitors yielded threshold values for DPP-IV inhibition that were slightly lower than the ones used in the original VS workflow [1] (*ET_pb* ≥0.468 and ST ≥0.237 instead of *ET_pb* ≥0.623 and ST ≥0.244), probably because the original threshold values were obtained exclusively using potent DPP-IV inhibitors (*i.e.*, IC_50_≤10 nM); in this study, this condition was relaxed slightly. After rescoring with EON, only those NPs with at least one conformation with *ET_pb* ≥0.468 and ST ≥0.237 relative to 3C45’s inhibitor were considered to be DPP-IV inhibitor candidates.

### Structural Similarity Analysis

The molecules that survived the electrostatics/shape similarity filter were merged with 2,342 known DPP-IV inhibitors obtained from the BindingDB database [64] and then clustered using Canvas v1.2 (Schrödinger LLC., Portland, USA; http://www.schrodinger.com). MOLPRINT2D fingerprints [65], using a fingerprint precision of 32 bits, were calculated for each molecule, and hierarchical clustering, based on Tanimoto similarities, was subsequently obtained. The number of clusters obtained was defined using the Kelley criterion [66], corresponding to a Tanimoto coefficient of 0.775 in this case.

## Supporting Information

Table S1 **Natural extracts with reported antidiabetic activity that contain molecules that were predicted to be DPP-IV inhibitors by our VS protocol.** The first column shows the 2D structure of each molecule and, when available, the corresponding common name and/or CAS number. The second column shows the number of the cluster in which the corresponding molecule was classified when its structure was compared with those of a group of 2,342 known DPP-IV inhibitors. The third column shows the scientific name of one of the sources in which the antidiabetic activity has been reported (rows in that table are alphabetically sorted based on this column). Bibliographic references for each molecule are divided into three columns in which (a) the first column presents papers that describe the purification of the molecule from the corresponding extract; (b) the second column lists papers that describe the antidiabetic activity of the corresponding extract; and (c) the third column lists papers, when available, that describe the antidiabetic activity of the corresponding molecule or one that is very similar to it.
(DOC)

Table S2 **Natural extracts with no described antidiabetic activity (but from the same** ***genus*** **as plants with extracts with described antidiabetic activity) that contain molecules that are predicted to be DPP-IV inhibitors by our VS protocol.** The first column shows the 2D structure of each molecule and, when available, the corresponding common name or CAS number. The second column shows the number of the cluster in which the corresponding molecule was classified when its structure was compared with those of a group of 2,342 known DPP-IV inhibitors. The third column lists the source from which the VS hits have been purified (rows in that table are alphabetically sorted based on this column). The fourth column lists the papers that describe the purification of the each molecule from the corresponding extract. The fifth column shows which are the extracts from the same *genus* where the antidiabetic activity has been described. Finally, the last column lists papers that describe the antidiabetic activity of the corresponding extract.
(DOC)
