# Supplementary material for: Identification of Novel Human Dipeptidyl Peptidase-IV Inhibitors of Natural Origin (Part II): In Silico Prediction in Antidiabetic Extracts
Source: PLoS One. 2012 Sep 21;7(9):e44972. doi: 10.1371/journal.pone.0044972 (PMC3448616; doi:10.1371/journal.pone.0044972)
Supplement: Table S1 — Natural extracts with reported antidiabetic activity that contain molecules that were predicted to be DPP-IV inhibitors by our VS protocol. The first column shows the 2D structure of each molecule and, when available, the corresponding common name and/or CAS number. The second column shows the number of the cluster in which the corresponding molecule was classified when its structure was compared with those of a group of 2,342 known DPP-IV inhibitors. The third column shows the scientific name of one of the sources in which the antidiabetic activity has been reported (rows in that table are alphabetically sorted based on this column). Bibliographic references for each molecule are divided into three columns in which (a) the first column presents papers that describe the purification of the molecule from the corresponding extract; (b) the second column lists papers that describe the antidiabetic activity of the corresponding extract; and (c) the third column lists papers, when available, that describe the antidiabetic activity of the corresponding molecule or one that is very similar to it. (DOC) [file pone.0044972.s001.doc]

**Table S1.** Natural extracts with reported antidiabetic activity that contain molecules that were predicted to be DPP-IV inhibitors by our VS protocol.

| **Molecule CAS number or Name** | **Cluster** | **Extract** | **Ref. Isolation Molecule from Extract** | **Ref. Antidiabetic Extract** | **Ref. Antidiabetic Molecule** |
| --- | --- | --- | --- | --- | --- |
| 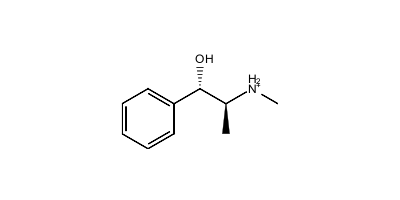  (+)-pseudoephedrine (90-82-4) | 86 | *Ephedra*  *alata* | [34] | [38] | [33] |
| 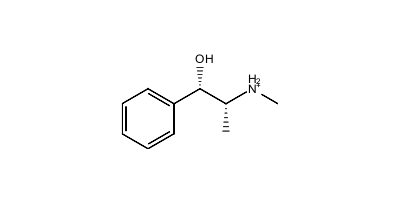  (-)-ephedrine (299-42-3) | 86 | *Ephedra distachya* | [35] | [33] | [33] |
| 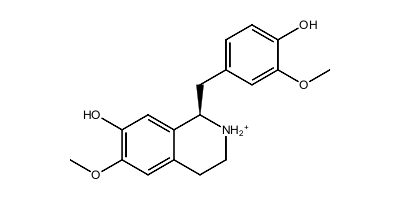  N-nororientalin (29079-44-5) | 89 | *Erythrina variegata* | [22] | [23] | [24-26] |
| 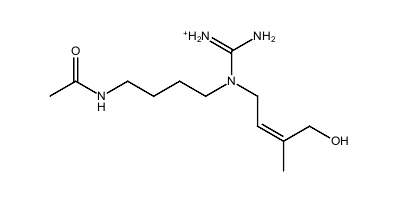  hydroxysmirnovine | 31 | *Galega orientalis* | [52] | [67] |  |
| 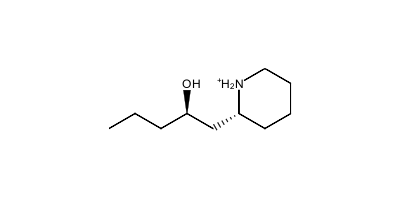  (-)-halosaline (26648-71-5) | 79 | *Haloxylon salicornicum* | [53] | [38] |  |
| 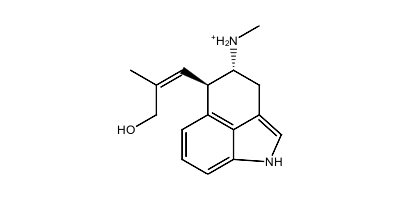  isochanoclavin-(I) (1150-43-2) | 17 | *Pennisetum typhoideum* | [54] | [68] |  |
| 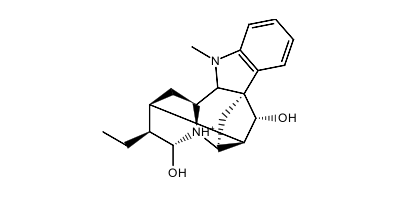  ajmaline (509-37-5) | 78 | *Rauwolfia serpentina* | [46] | [40] |  |
| 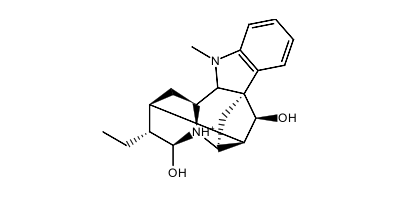  isosandwichine (509-37-5) | 78 | *Rauwolfia vomitoria* | [69] | [39] |  |
| 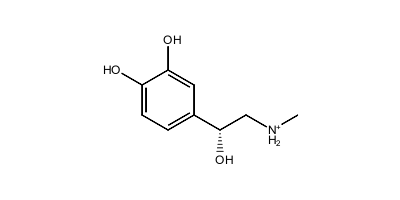  epinephrine (51-43-4) | 86 | *Scoparia dulcis* | [70] | [31] | [30] |
| 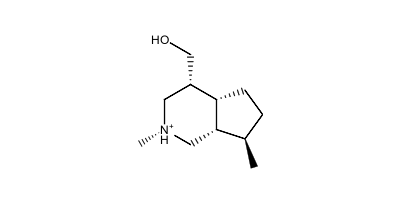  tecostanine | 98 | *Tecoma*  *stans* | [71] | [29] | [28] |
| 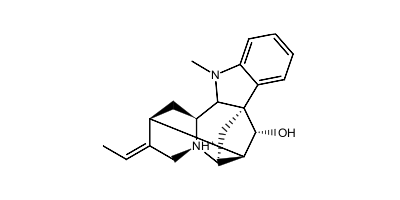  serpinine (509-38-6) | 78 | *Vinca*  *major* | [54] | [47] |  |
| 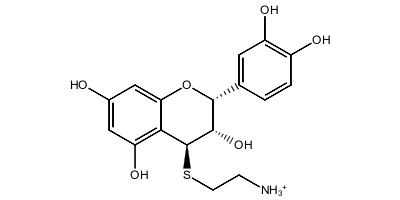  epicatechin derivate | 91 | *Vitis*  *vinifera* | [72] | [48] | [50] |

The first column shows the 2D structure of each molecule and, when available, the corresponding common name and/or CAS number. The second column shows the number of the cluster in which the corresponding molecule was classified when its structure was compared with those of a group of 2,342 known DPP-IV inhibitors. The third column shows the scientific name of one of the sources in which the antidiabetic activity has been reported (rows in that table are alphabetically sorted based on this column). Bibliographic references for each molecule are divided into three columns in which (a) the first column presents papers that describe the purification of the molecule from the corresponding extract; (b) the second column lists papers that describe the antidiabetic activity of the corresponding extract; and (c) the third column lists papers, when available, that describe the antidiabetic activity of the corresponding molecule or one that is very similar to it.
