# Supplementary material for: Identification of Novel Human Dipeptidyl Peptidase-IV Inhibitors of Natural Origin (Part II): In Silico Prediction in Antidiabetic Extracts
Source: PLoS One. 2012 Sep 21;7(9):e44972. doi: 10.1371/journal.pone.0044972 (PMC3448616; doi:10.1371/journal.pone.0044972)
Supplement: Table S2 — Natural extracts with no described antidiabetic activity (but from the same genus as plants with extracts with described antidiabetic activity) that contain molecules that are predicted to be DPP-IV inhibitors by our VS protocol. The first column shows the 2D structure of each molecule and, when available, the corresponding common name or CAS number. The second column shows the number of the cluster in which the corresponding molecule was classified when its structure was compared with those of a group of 2,342 known DPP-IV inhibitors. The third column lists the source from which the VS hits have been purified (rows in that table are alphabetically sorted based on this column). The fourth column lists the papers that describe the purification of the each molecule from the corresponding extract. The fifth column shows which are the extracts from the same genus where the antidiabetic activity has been described. Finally, the last column lists papers that describe the antidiabetic activity of the corresponding extract. (DOC) [file pone.0044972.s002.doc]

**Table S2.** Natural extracts with no described antidiabetic activity (but from the same *genus* as plants with extracts with described antidiabetic activity) that contain molecules that are predicted to be DPP-IV inhibitors by our VS protocol.

| **Molecule CAS number or Name** | **Cluster** | **Extract** | **Ref. Isolation Molecule from Extract** | **Antidiabetic Extract** | **Ref. Antidiabetic Extract** |
| --- | --- | --- | --- | --- | --- |
| 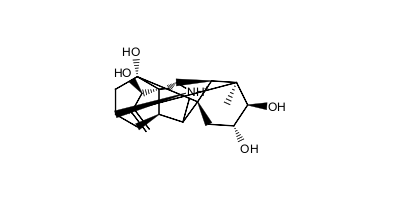  30373-79-6 | 96 | *Aconitum*  *japonicum* | [73] | *Aconitum carmichaelii* | [74] |
|  |  |  |  | *Aconitum moschatum* | [2] |
|  |  |  |  | *Aconitum violaceum* | [2] |
| 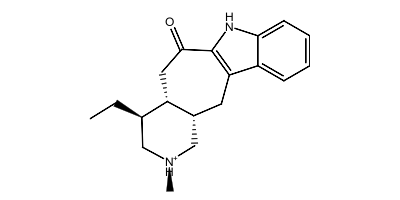  episilicine | 21 | *Ervatamia*  *officinalis* | [75] | *Ervatamia microphylla* | [55] |
| 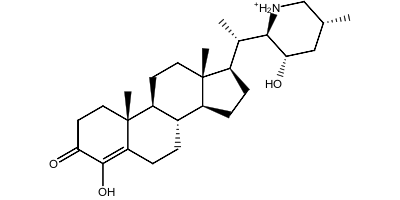  solanudine | 97 | *Solanum*  *nudum* | [76] | *Solanum lycocarpum* | [56] |
|  |  |  |  | *Solanum*  *nigrum* | [57] |
| 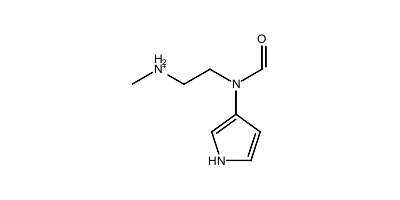 | 2 | *Solanum*  *sodomaeum* | [77] | *Solanum xanthocarpum* | [58] |
| 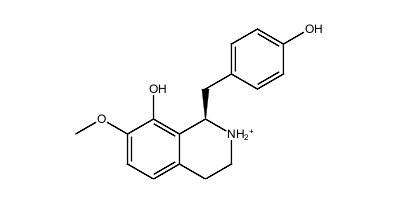  norjuziphine | 89 | *Stephania cepharantha* | [78] | *Stephania hernandifolia* | [59] |
|  |  |  |  | *Stephania*  *glabra* | [60] |
|  |  |  |  | *Stephania tetrandra* | [61] |
| 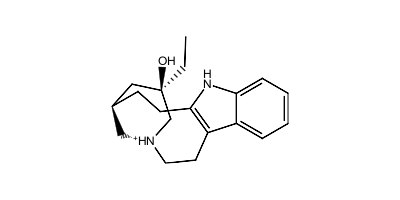  19637-92-4 | 20 | *Tabernaemontana eglandulosa* | [79] | *Tabernaemontana divaricata* | [55] |

The first column shows the 2D structure of each molecule and, when available, the corresponding common name or CAS number. The second column shows the number of the cluster in which the corresponding molecule was classified when its structure was compared with those of a group of 2,342 known DPP-IV inhibitors. The third column lists the source from which the VS hits have been purified (rows in that table are alphabetically sorted based on this column). The fourth column lists the papers that describe the purification of the each molecule from the corresponding extract. The fifth column shows which are the extracts from the same *genus* where the antidiabetic activity has been described. Finally, the last column lists papers that describe the antidiabetic activity of the corresponding extract.
